# News and Coming Events

**Published:** 1908-07-04

**Authors:** 


### News and Coming Events.

Dr. John Cameron, M.D., D.Sc., M.R.C.S., F.R.S.E., has been appointed Lecturer on Anatomy in the Medical School of the Middlesex Hospital.

The treasurers of the Middlesex Hospital have received from Mr. Andrew Carnegie a donation of £100 in aid of the fund for Cancer Research at the Hospital.

Dr. Laurence Humphry has been reappointed Assessor to the Regius Professor of Physic at the University of Cambridge for the ensuing academical year.

The three days' bazaar opened by Princess Henry of Battenberg realised £1,300 towards the debt of £8,000 on the Queen's Hospital for Children, Hackney Road.

Up to the time of going to press with this number of The Hospital, the total of the Hospital Sunday Fund, resulting from collections in churches and chapels, was more than £20,000.

Dr William Robert Smith, M.D., V.D., Brigade Surgeon-Lieutenant-Colonel (Honorary Surgeon-Colonel), 3rd London Volunteer Infantry Brigade, has been appointed Deputy-Lieutenant of the County of London, dated June 18.

By invitation of Major-General Lord Cheylesmore and the committee of management, the Brompton Hospital Sanatorium, Frimley, will be open for inspection, with the patients at work, this afternoon (Saturday, July 4) at 3 o'clock.

The annual meeting of the Association for the Oral Instruction of the Deaf and Dumb will be held at the Portman Rooms, Baker Street, W., on Monday, July 6, at 4.30 p.m., when a short illustration of the system will be given. Leopold de Rothschild, Esq., will preside.

On the occasion of the 100th anniversary of the foundation of the Physico-Medical Society of Vienna, the following appointments, among others, have been made:—Sir Victor Horsley to be honorary Ph.D.; to be corresponding members of the society:—Dr. J. Loeb, Professor of Physiology at the University of California, Dr. C. S. Minot, of Boston, U.S.A., Dr. E. Rutherford, F.R.S., of Manchester University, and Dr. C. S. Sherrington, F.R.S., of Liverpool University.

At a meeting of the Royal College of Physicians of Edinburgh, on June 23, the vacancy in the office of President created by the death of the late Dr. C. E. Underhill was filled by the appointment of the Vice-President, Dr. John Playfair, for the remainder of the current academic year.

Colonel Sir David Bruce, who will be in charge of the new commission to proceed to East Africa to investigate sleeping sickness, will be accompanied by Captain A. E. Hamerton, R.A.M.C., and not Captain Hamilton, as was stated, on the authority of Reuter's Agency, in our last issue.

A performance in aid of the Royal Waterloo Hospital for Children and Women was given at the Royal Court Theatre on the evening of July 1, under the immediate patronage of Princess Alexander of Teck and Prince Alexander of Teck. The pieces presented were "Petticoat Perfidy" and "Cousin Kate."

The Gulstonian lectures for 1909 at the Royal College of Physicians of London will be delivered by Dr. A. E. Russell; the Lumleian lectures by Dr. Norman Moore; the Fitz-Patrick lectures by Sir T. Clifford Allbutt; the Oliver-Sharpey lectures by Professor C. Scott Sherrington; and the Croonian lectures for 1910 by Dr. Arthur Gamgee.

A special matinée has been arranged to take place at the Lyceum Theatre on Tuesday, July 14, in aid of King's College Hospital Removal Fund. Most of the leading actors and actresses in London have offered their services. Her Majesty the Queen and their Royal Highnesses the Duke and Duchess of Connaught have taken boxes for the occasion.

An appeal is being made by the committee of University College Hospital for funds, in which it is pointed out that £19,000 is required each year in voluntary contributions to cover the deficit between annual expenditure and reliable income. The committee say that they have recently been compelled to dispose of £4,000 of their small available funded property in order to pay overdue accounts of 1907. The financial outlook is more gloomy than it has been for years, and substantial and immediate help is absolutely imperative in order that the number of beds may not be reduced and the wide-reaching usefulness of the charity curtailed.

The annual conversazione and exhibition of medical and surgical appliances of the West London Medico-Chirurgical Society was held on June 26 at the Kensington Town Hall. There are about 700 members, and many of these were present with their friends. After the reception by the president, Mr. R. Lake, Sir William Whitla delivered the Cavendish lecture, selecting for his subject "The Ætiology of Phthisis."

The first meeting of the Socialist Medical League was held on June 25 under the chairmanship of Dr. A. Salter, L.C.C. Over seventy medical practitioners have become members of the League. Membership is limited to registered British medical practitioners and dentists who accept the principles of Socialism. It was arranged to hold a yearly meeting during the annual meeting of the British Medical Association and in the same town. Dr. A. Salter was elected Chairman, Dr. Williams Treasurer, and Dr. Eder Secretary.

A committee has been appointed by the Council of the Royal College of Surgeons of England, to combine with any committee which may be appointed by the Royal College of Physicians of London for the same purpose, to consider and draft a scheme which, if approved by the Colleges, should be submitted to the Senate of the University of London, with the object of establishing a system of conjoint examination for the degrees of M.B. and B.S., and the diplomas of L.R.C.P. London. and M.R.C.S. Eng.

H.R.H. Princess Alexander of Teck opened, on June 30, the extension of the Nurses' Home and the new residential college for students in connection with Queen Charlotte's Lying-in Hospital, Marylebone Road. Her Royal Highness was received at the hospital by Viscount Portman, the President, who was accompanied by Viscountess Portman, and there were presented to her Sir Samuel Scott, M.P., Chairman of the Committee of Management; Mr. H. A. Harben, Vice-Chairman; Major McMahon, Chairman of the Building Committee; Dr. Pollock and Dr. Gow, representing the medical staff; Mr. F. W. Hunt, honorary architect; and Mr. A. Watts, secretary. Her Royal Highness, after declaring the building open, made an inspection of the new premises and visited some of the wards

Lord Plymouth, one of the treasurers, distributed the prizes on June 24 to the successful students at St. George's Hospital. The senior physician, Dr. Rolleston, referred to the close connection between the hospital and the school, and to the interest which the governors had always taken in medical education. Lord Plymouth said that he was glad to see that an endowment fund had been successfully initiated at St. George's Hospital, as it placed the school in an advantageous position. He expressed the opinion that this fund ought to be greatly increased in order that a settled income might be derived from it which would enable the hospital and school to give not only a good education in all the branches of medical science, but to promote research. The senior surgeon, Mr. Clinton Dent, in proposing a vote of thanks to Lord Plymouth, said that the governors and the school were closely related; without the school the work done by the hospital would inevitably deteriorate. There might come a time when the present state of affairs would be at an end, when hospitals would be rate aided and State governed, but when that time came the work now done by the hospitals would be three times as costly as it is now, and one-third as efficient. He hoped that St. George's Hospital would work in the future as it has in the past.

The Guy's Hospital Biennial Dinner, 1908, will be held on Wednesday, July 8, at 7 p.m., at the Hôtel Cecil, under the chairmanship of Charters J. Symonds, Esq., M.S.

The eleventh French Medical Congress will take place this year at Geneva from September 3 to 5. Medical men can obtain of the management of the Paris, Lyons and Mediterranean Railway (88 Rue St. Lazare), tickets from Paris to Geneva at a reduction in cost of 50 per cent.

The portrait of Prince Edward to be placed in the children's building of the Hunstanton Convalescent Home has been approved by the Princess of Wales, and her Royal Highness has sent a case of toys for the children in the Prince Edward Home. The committee have accepted the gift of a cot by Mrs. Isabel M. Lowe in memory of her husband, the late Dr. John Lowe, of Lynn, for thirty years and until his death honorary consulting physician to the Home.

Dr. Osler, Regius Professor of Medicine in Oxford University, has consented to stand as non-party candidate for the Lord Rectorship of the University of Edinburgh. The political candidates already adopted are Mr. Churchill, M.P., and Mr. Wyndham, M.P., so that there will be a three-cornered fight. In regard to the forthcoming contest, according to the *Times*, it is said that Mr. Wyndham's name has been received coldly by the Unionists, and that on account of his lack of University training many Liberals refuse to support the President of the Board of Trade. The medical vote, 1,400 strong, is expected to go almost completely to Dr. Osler. The election is in November.

The Jubilee Festival dinner of the Royal Dental Hospital of London was held on June 25 at the Hôtel Cecil. Sir Frederick Treves, who presided, said in giving the toast of "The King" that his Majesty had headed the list of subscriptions for the evening with a gift of £50. Inspector-General James Porter, Director-General of the Naval Medical Department, and Surgeon-General Sir A. Keogh, Director-General of the Army Medical Service, both spoke highly of the new system of dental inspection and repair now carried out in the Services. The Chairman said the hospital was appealing for £47,000. Among those who also spoke were Sir R. Douglas Powell and Sir James Crichton Browne. During the evening subscriptions were announced amounting to about £3,000.

At Middlesex Hospital, on June 29, Dr. William Cayley, consulting physician to the hospital, unveiled in the corridor a mural memorial to the late Mr. Edward Ashby Fardon, for twenty-eight years resident medical officer there. The memorial takes the form of a medallion in bronze, with the name above, and beneath it the inscription: "Resident medical officer, 1879-1907. An able administrator, a faithful servant of the hospital, a much-loved physician." There will also be a gold, a silver, and a bronze medal with a portrait of Mr. Fardon on the obverse, to be awarded to three nurses every year. At a meeting held in the board room before the unveiling ceremony Dr. Cayley spoke of the many services rendered to the hospital by Mr. Fardon, of the practical interest he had taken in the many improvements and additions to the hospital, and the time he had devoted to the welfare and advancement of the nursing staff. Major-Gen. Lord Cheylesmore, Chairman of the weekly board, accepted on its behalf models of the medals to be awarded to the nurses. He referred to Mr. Fardon's work at the hospital, to which, he said, his life had been devoted, and added that he was a colleague beloved and admired by all. No hospital ever had a better resident medical officer, and by his death they had sustained an irreparable loss.

